# A Novel MRI-Based Finite Element Modeling Method for Calculation of Myocardial Ischemia Effect in Patients With Functional Mitral Regurgitation

**DOI:** 10.3389/fphys.2020.00158

**Published:** 2020-03-13

**Authors:** Yue Zhang, Vicky Y. Wang, Ashley E. Morgan, Jiwon Kim, Liang Ge, Julius M. Guccione, Jonathan W. Weinsaft, Mark B. Ratcliffe

**Affiliations:** ^1^San Francisco Veterans Affairs Medical Center, San Francisco, CA, United States; ^2^Department of Surgery, University of California, San Francisco, San Francisco, CA, United States; ^3^Department of Bioengineering, University of California, San Francisco, San Francisco, CA, United States; ^4^Department of Medicine, Weill Cornell Medicine, New York, NY, United States

**Keywords:** myocardial ischemia, myocardial infarction, coronary artery disease, coronary artery bypass, mitral valve insufficiency, inverse finite element analysis, computer simulation

## Abstract

**Background:**

Functional Mitral Regurgitation (FMR) associated with coronary artery disease affects nearly 3 million patients in the United States. Both myocardial infarction (MI) and ischemia contribute to FMR development but uncertainty as to which patients will respond to revascularization (REVASC) of ischemia alone prevents rational decision making about FMR therapy. The aim of this study was to create patient-specific cardiac MRI (CMR) informed finite element (FE) models of the left ventricle (LV), calculate regional LV systolic contractility and then use optimized systolic material properties to simulate the effect of revascularization (virtual REVASC).

**Methods:**

We describe a novel FE method able to predict the effect of myocardial ischemia on regional LV function. CMR was obtained in five patients with multi-vessel coronary disease and FMR before and 3 months after percutaneous REVASC and a single healthy volunteer. Patient-specific FE models were created and divided into 17 sectors where the systolic contractility parameter, *Tmax* of each sector was a function of regional stress perfusion (SP-CMR) and myocardial infarction (LGE-CMR) scores. Sector-specific circumferential and longitudinal end-systolic strain and LV volume from CSPAMM were used in a formal optimization to determine the sector based myocardial contractility, *Tmax* and ischemia effect, α. Virtual REVASC was simulated by setting α to zero.

**Results:**

The FE optimization successfully converged with good agreement between calculated and experimental end-systolic strain and LV volumes. Specifically, the optimized *T*_*max*_ for the healthy myocardium for five patients and the volunteer was 495.1, 336.8, 173.5, 227.9, 401.4, and 218.9 kPa. The optimized α was found to be 1.0, 0.44, and 0.08 for Patients 1, 2, and 3, and 0 for Patients 4 and 5. The calculated average of radial strain for Patients 1, 2, and 3 at baseline and after virtual REVASC was 0.23 and 0.25, respectively.

**Conclusion:**

We developed a novel computational method able to predict the effect of myocardial ischemia in patients with FMR. This method can be used to predict the effect of ischemia on the regional myocardium and promises to facilitate better understanding of FMR response to REVASC.

## Introduction

Functional mitral regurgitation (FMR) associated with coronary artery disease (CAD) is a leading cause of valvular heart disease. FMR occurs in 1.2 to 2.1 million patients with CAD (Gorman et al., 2003) in whom it doubles the risk of heart failure and death (Aronson et al., 2006).

Both myocardial infarction (MI) and ischemia contribute to FMR development. Specifically, creation of inferior MI in large animals leads to left ventricular (LV) remodeling, papillary muscle displacement, restriction of mitral leaflets and subsequent development of FMR (Gorman et al., 1995; Liel-Cohen et al., 2000). Conversely, the evidence for ischemia comes from clinical studies where in approximately half of patients, FMR improves with coronary revascularization (REVASC), whereas in the remainder FMR persists or worsens (Aklog et al., 2001; Penicka et al., 2009; Kang et al., 2011). Uncertainty as to which patients will respond to REVASC impedes rational decision-making regarding FMR management.

Cardiac magnetic resonance imaging (CMR) allows LV remodeling, tissue characteristics including MI and non-ischemic fibrosis, ischemia and regional contractile function to be assessed with high precision. Cine-CMR has been employed as a reference standard for measuring LV size and function (Codella et al., 2008, 2012; Janik et al., 2008). Stress perfusion CMR (SP-CMR) has been shown to provide high diagnostic accuracy for ischemia as can occur with obstructive CAD (Klem et al., 2006). Late gadolinium enhancement CMR (LGE-CMR) provides near exact agreement with histopathology evidenced MI (Kim et al., 1999, 2000) and CMR with non-invasive tags CSPAMM-CMR has been widely used to measure systolic myocardial deformation and strain (Ryf et al., 2002).

The relationship between MI fibrosis, ischemia and function and recovery of function after REVASC in chronically ischemic hibernating myocardium in which myocardial metabolism and contractile function are down-regulated (Wijns et al., 1998) is complex (Canty and Suzuki, 2012). In that regard, we recently measured MI fibrosis, stress perfusion and regional strain with CMR in patients with FMR associated with CAD (Morgan et al., 2018). Briefly, CSPAMM-CMR measured circumferential and longitudinal strain were modestly reduced in sectors without MI or ischemia when compared to volunteers and then decreased in an approximately linearly fashion as the amount of MI fibrosis and ischemia increased (Morgan et al., 2018). In addition, chronically ischemic hibernating myocardium can partially recover function after surgical or percutaneous REVASC (Kim et al., 2000; Hocum Stone et al., 2017). However, fewer than 10% of dysfunctional segments with LGE hyperenhancement greater than 50% recover function (Kim et al., 2000).

Finite element (FE) modeling has the unique potential to (1) determine the effect of ischemia in FMR associated with CAD and to then (2) simulate the effect of REVASC. Prior FE modeling studies by our group (Walker et al., 2005; Wenk et al., 2011) and others (Bogen et al., 1980; Genet et al., 2015) have focused on the impact of MI but without incorporation of ischemia effect. Most previous FE modeling studies of the LV with ischemia have focused on arrhythmogenesis (Wang et al., 2013; Mendonca Costa et al., 2018) without incorporation of ischemia effect on LV function. There have been FE modeling studies of ischemia effect on regional LV function in which models were based on an idealized LV shape (Bovendeerd et al., 1996), cardiac imaging (XCAT) phantom (Veress et al., 2015) and physical measurements in an excised large animal heart (Mazhari et al., 2000). However, in those studies, regions of ischemia were assumed to have little (Mazhari et al., 2000) or no (Bovendeerd et al., 1996; Veress et al., 2015) regional contractile function. To date, FE models that incorporate CMR-based tissue characteristics to determine regional contractility have not been described.

This study describes a novel FE method able to predict the effect of myocardial ischemia on regional LV function. The method leverages CMR imaging data obtained from patients with multivessel CAD and FMR before and after percutaneous REVASC. It includes patient-specific FE models in which the regional systolic contractility parameter is a function of regional CMR-based tissue characteristics including stress perfusion and MI. The aim of the study is to calculate systolic material properties of ischemic myocardium and then use optimized systolic material properties to simulate the effect of REVASC (virtual REVASC).

## Materials and Methods

Five patients were selected from a larger group of patients prospectively enrolled in a protocol entitled “CMR Myocardial Tissue Based Prediction of Ischemic MR Revascularization Response” at Weill Cornell Medical College (New York, NY, United States) examining REVASC effect in patients with FMR associated with CAD. The protocol included CMR and transthoracic echocardiography before and 3 months after percutaneous revascularization (PCI). One healthy volunteer without cardiovascular risk factors was also studied. The Cornell Institutional Review Board approved this study, and written informed consent was obtained at time of enrollment.

### Computational Modeling Pipeline

**FIGURE 1:**
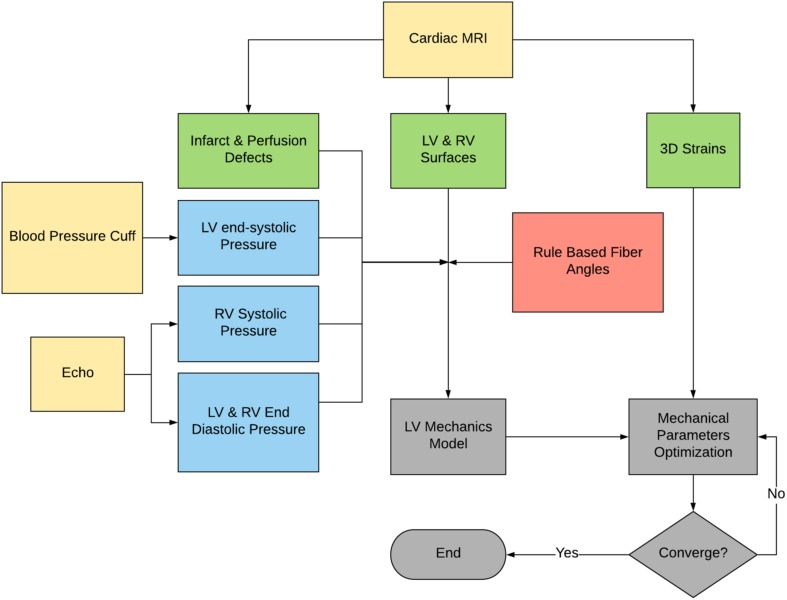
Flowchart of the proposed method. Note that the raw data are in yellow, the derived pressure in blue, the CMR-derived geometry and strains in green, and the FE modeling process in gray.

The proposed method is summarized in Figure 1. The CMR images were used to create LV and right ventricular (RV) surfaces (see section “Image Analysis”), to calculate 3D regional strains (see section “Constitutive Laws”), and to configure LV mechanical parameters (see section “Sector-Based Myocardial Material Parameters”). LV mechanics models were constructed using the patient-specific LV and RV surfaces, LV and RV pressures, rule-based fiber angles, diastolic and systolic myocardial constitutive relationships and LV myocardial material parameters. A formal parameter optimization framework was then constructed based on the LV mechanics models and the *in vivo* sector-specific circumferential and longitudinal end-systolic strain and LV volume.

### Image Acquisition

**FIGURE 2:**
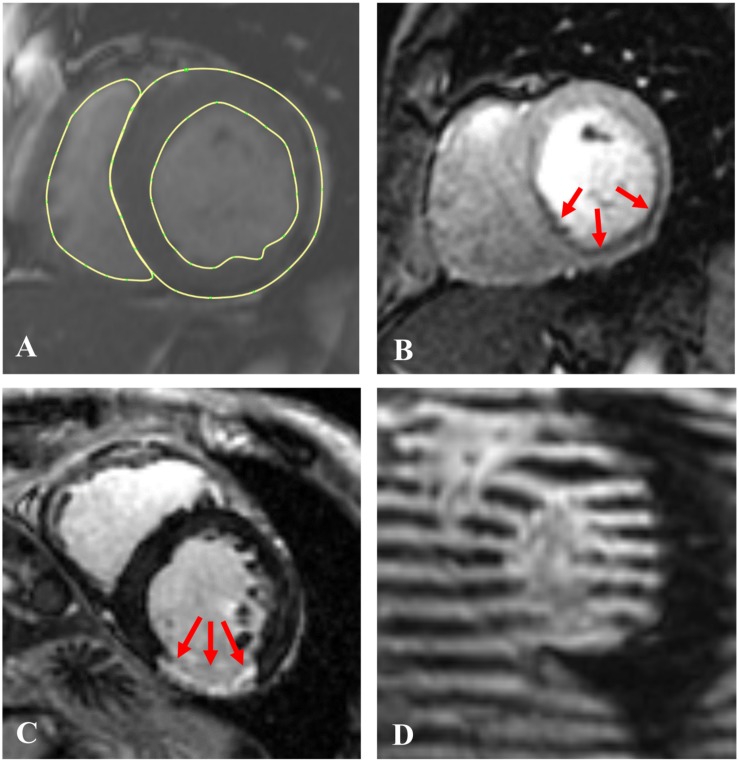
Examples of the 4 CMR image sequences used in this study: **(A)** Short axis Cine-CMR with LV epicardial and endocardial and RV endocardial contours; **(B)** A stress perfusion image with arrows indicating the ischemic region; **(C)** A late gadolinium enhancement (LGE-CMR) image with red arrows indicating the infarcted region, and **(D)** A short axis tagged (CSPAMM) image.

CMR was performed with a 3.0 Tesla MRI scanner (General Electric, Waukesha, WI, United States) (Morgan et al., 2018) both before and 3 months after PCI. CMR included 4 pulse sequences. Specifically, Cine-CMR was performed with a steady-state free precession pulse sequence. Images were acquired in contiguous LV short axis and standard (2-, 3- and 4-chamber) long axis orientations (Figure 2A). SP-CMR was assessed in accordance with established methods previously validated by members of our group (Klem et al., 2006; Heitner et al., 2019). In brief, pharmacologic stress was induced with regadenoson (0.4 mg), during which gadolinium was infused (0.1 mmol/kg) and LV short axis images (4–5, evenly distributed from base-apex) were acquired using a gradient echo pulse sequence (Figure 2B). Perfusion CMR was repeated 5-min thereafter under baseline (non-stress) conditions. Myocardial infarction (LGE-CMR) was assessed using an inversion recovery pulse sequence, which was acquired 10–30 min post-gadolinium (0.2 mmol/kg) infusion and acquired in spatial orientations matched to Cine-CMR (Figure 2C). CSPAMM in contiguous LV short and long axis slices (8 mm tag spacing, 10 mm slice thickness, no gap) was performed to measure myocardial deformation and strain (Figure 2D).

### Image Analysis

#### LV and RV Segmentation

Left ventricular short and long axis and RV short axis Cine-CMR images were contoured at LV early diastolic filling (EDF; MeVisLab, version 2.7.1, Bremen, DE) where the EDF phase was defined as the time step immediately prior to opening of the mitral valve. The LV and RV contours included the papillary muscle bases but excluded the trabeculae (Figure 2A).

To eliminate short axis image alignment breathing artifacts, an LV surface was created based on the three epicardial long axis contours. LV short axis contours were moved in plane so that the contour centroid was over the centroid of the corresponding plane from the long axis surface. After alignment, LV epicardial and endocardial and RV endocardial surfaces were created using the aligned short axis contours.

#### Scoring of Cine-, SP- and LGE-CMR Images

Left ventricular function, MI scar and stress perfusion were localized using the AHA/ACC 17 segment model (Cerqueira et al., 2002). LV wall thickening was measured on Cine-CMR using a 5-point scale (0 = normal (at least 50% wall thickening), 1 = mild hypokinesis, 2 = moderate hypokinesis, 3 = akinesis (no wall thickening), 4 = dyskinesis).

Myocardial infarction was identified on LGE-CMR, for which transmural extent was graded using a 5-point segmental scale (0 = no hyperenhancement; 1 = 1–25%; 2 = 26–50%; 3 = 51–75%; 4 = 76–100%) (Sievers et al., 2007). LGE in non-coronary arterial patterns (mid-wall or epicardial) was not included in MI analyses.

Ischemia was assessed semi-quantitatively on SP-CMR based on peak myocardial signal intensity during first-pass gadolinium infusion. Perfusion deficits were defined as persistent hypo-enhancement on ≥4 consecutive heart beats (Klem et al., 2006) and the severity of stress perfusion defects was graded semi-quantitatively using an established 4-point scale (0 = absent, 1 = mild, 2 = moderate, 3 = severe) based on the magnitude of hypo-enhancement (Kim et al., 2019).

#### CMR-Measured Strains

Regional 3D circumferential (*E*_*cc*_) and longitudinal (*E*_*ll*_) strain for each sector were calculated from CSPAMM images. The HARmonic Phase (HARP) method developed by Osman et al. (2000) was implemented. Implementation in MeVisLab has been previously reported (Morgan et al., 2018). Please note that all the strain presented in this study is LV end-systolic strain.

### LV FE Model Generation

**FIGURE 3:**
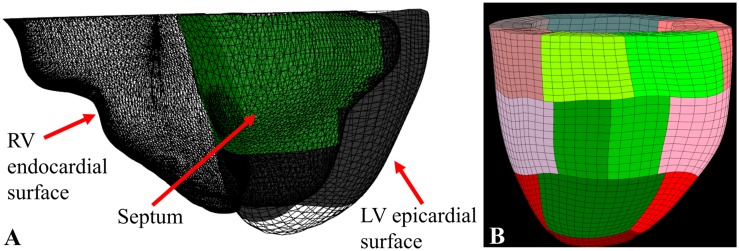
**(A)** RV endocardial surface (triangle mesh), LV epicardial surface (hexahedral mesh), and the septum (in green) determined using a ray-casting method; **(B)** the final 17 sector FE model with the septal sectors in green.

Using LV epicardial and endocardial surfaces as input, TruGrid (XYZ Scientific Applications, Inc., Pleasant Hill, CA, United States) was used to create patient-specific meshes of the LV composed of hexahedral elements as shown in Figure 3. Meshes with different density were created and optimal mesh density was determined as a function of parameter calculation accuracy and calculation time.

Each element was assigned a material direction with a rule-based fiber angle method (Bayer et al., 2012) where the myofiber helix angle varying transmurally from 60° at the endocardium to −60° at the epicardium.

Custom software (C#, Visual Studio 2017, Microsoft, Redmond, WA, United States) that included a ray casting method (Möller and Trumbore, 1997) to locate the interventricular septum (Figure 3A) was used to break the mesh into 17 AHA sectors (Cerqueira et al., 2002; Figure 3B)^1^.

### Loading and Boundary Condition

LV pressure (LVP) at end systole was obtained from a blood pressure cuff and LVP at end-diastole (ED) and RV end-diastolic and systolic pressures were estimated from concomitant transthoracic echocardiography data (Ommen et al., 2000; Table 1). Patient-specific LV pressure was applied to the LV endocardial surface and RV pressure was applied to the LV septal epicardial surface.

**TABLE 1:** Patient-specific LV and RV pressures and LV ejection fraction (EF).

	**Patient 1**	**Patient 2**	**Patient 3**	**Patient 4**	**Patient 5**	**Volunteer**
LV EDP [mmHg]	20	20	20	20	10	10
LV ESP [mmHg]	121	140	100	134	140	120
RV EDP [mmHg]	3	8	8	3	8	8
RV ESP [mmHg]	23	28	23	20	40	25
Ejection fraction (%)	36	31	15	43	45	58

Note that these models contract from the apex to the base. Specifically, nodes at the LV base (top surface) were fixed in the valve plane (Z direction movement = 0) but able to slide in the *X* and *Y* directions during passive filling and systolic contraction.

### Constitutive Laws

Passive and active myocardial constitutive laws described by Guccione et al. (1991, 1993) were used in this study. Specifically, the passive myocardium is modeled by a strain energy function, *W*, that is anisotropic relative to the local fiber direction:

(1) $$W=0.5\,C\,\left(e^{Q}-1\right)$$

where

(2) $$Q=[b_{f}E_{11}^{2}+b_{t}\left(E_{22}^{2}+E_{33}^{2}+E_{23}^{2}+E_{32}^{2}\right)+$$

$$b_{f\,s}\left(E_{12}^{2}+E_{21}^{2}+E_{13}^{2}+E_{31}^{2}\right)]$$

*E*_*11*_ is fiber strain, *E*_*22*_ is cross-fiber in-plane strain, *E*_*33*_ is radial strain, *E*_*23*_ is shear strain in the transverse plane, and *E*_*12*_ and *E*_*13*_ are shear strain in the fiber-cross fiber and fiber-radial planes, respectively, and where b_*f*_ = 49.25, b_*t*_ = 19.25 and b_*fs*_ = 17.44 (Walker et al., 2005).

A time-varying elastance model is used to simulate the active contraction of cardiac muscles (Guccione et al., 2001). The time-varying elastance model has the following form:

(3) $$T_{0}=T_{m\,a\,x}\,\frac{C\,a_{0}^{2}}{C\,a_{0}^{2}+E\,C\,a_{50}^{2}}\,C_{t}$$

where *T*_*max*_ is the maximum isometric tension achieved at the longest sarcomere length and maximum peak intracellular calcium concentration(*Ca*_0_)_*max*_. *Ca*_*0*_ is the intracellular calcium concentration and *ECa*_*50*_ is length-dependent calcium sensitivity. *C*_*t *_ is a time-varying variable defined as follows:

(4) $$C_{t}=\frac{1}{2}\,\left[1-c\,o\,s\,\left(\varpi \right)\right]$$

where ϖ is a time and sarcomere length dependent variable that increases from 0 at the start of contraction to π during peak contraction and then decreases to 0 during diastole.

Material laws were implemented with a user-defined material subroutine in the explicit FE solver, LS-DYNA (Livermore Software Technology Corporation, Livermore, CA, United States).

### Sector-Based Myocardial Material Parameters

The LGE score was an integer from 0 to 4 and the SP score was an integer ranging from 0 to 3. In each case, a score of 0 represented the absence of disease. Further LGE and SP scoring details are described above (see section “Scoring of Cine-, SP- and LGE-CMR Images”). Passive stiffness, *C*, was determined using the following:

(5) $$C_{n}=C_{H}+9\cdot C_{H}\cdot \frac{L\,G\,E_{n}}{4}$$

where *C*_*n*_ represents the passive stiffness for each sector and *C*_*H*_ the stiffness of a “healthy” sector with LGE = 0. Note that when LGE = 4 (transmural MI), *C*_*n*_=10*C*_*H*_*whichis* consistent with our prior work (Wenk et al., 2011).

On the other hand, both LGE and SP were assumed to effect regional contractility, *Tmax* according to the following linear relationship:

(6) $$T\,m\,a\,x_{n}=T\,m\,a\,x_{H}\cdot \left(1-\frac{L\,G\,E_{n}}{4}\right)\cdot \left(1-\alpha \cdot \frac{S\,P_{n}}{3}\right)$$

where *Tmax*_*n*_ represents the contractility for each sector, *Tmax*_*H*_ the contractility for healthy sector with LGE and SP = 0, α the ischemia effect and *LGE*_*n*_ and *SP*_*n*_ the LGE and SP scores for each sector. It’s known that *Tmax*_*n*_≥0 and *LGE*_*n*_ is ∋[0, 4] and *SP*_*n*_ is ∋[0, 3] then it can be determined α∋[0, 1]. Since we assumed that the LGE and SP scores were 0 for the healthy volunteer, *C*_*n*_ and *Tmax*_*n*_ for the volunteer were equal to *C*_*H*_ and *Tmax*_*H*_, respectively.

### Model Optimization

A formal optimization of *C*_*H*_,*Tmax*_*H*_ and α were performed where the objective function for the optimization was taken to be the mean-squared errors (MSE) (Guccione et al., 2001). *C*_*H*_ was determined such that the FE model predicted LV ED volume matched the patient-specific *in vivo* measured volume. *Tmax*_*H*_ was estimated by minimizing the MSE between FE model-predicted and *in vivo* MRI-measured end-systolic longitudinal and circumferential strains and the LV end-systolic volume. The goal of the optimization is to minimize the MSE as follows:

O⁢b⁢j⁢e⁢c⁢t⁢i⁢v⁢e⁢f⁢u⁢n⁢c⁢t⁢i⁢o⁢n

$$=\frac{1}{2\,N}\,\left(\sum_{n=1}^{N}\left({\left(E_{c\,c,n}-{\bar{E}}_{c\,c,n}\right)}^{2}+{\left(E_{l\,l,n}-{\bar{E}}_{l\,l,n}\right)}^{2}\right)\right)$$

(7) $$\;+W\,{\left(\frac{V_{E\,S}-{\bar{V}}_{E\,S}}{{\bar{V}}_{E\,S}}\right)}^{2},$$

where *n* is the *in vivo* average strain at each sector (note the apex sector was excluded so *n* = 16), *E*_*cc,n*_ the calculated FE circumferential strain, *E*_*ll,n*_ the calculated longitudinal strain, *V*_*ES*_ the LV end-systolic volume and *W* is the weight applied to the volume term. The overbar represents the experimental *in vivo* measurements.

*W* = 10 was applied to the volume term to make the strain and volume effects more balanced. For each case, the *Tmax*_*H*_ was initially = 350 kPa. α was initially = 0.5 in the FMR patient models and = 0 in the healthy volunteer model. No constraints were applied to either *Tmax*_*H*_*or V*_*ES*_.

### Mesh Convergence Study

A mesh convergence study was performed on models of patients 2 and 5 to find the minimum number of elements needed to obtain stable calculations of *C*_*H*_, *Tmax*_*H*_, and α within the fastest computation time. Four sets of meshes with different density were created with the transmural elements from 3 to 4, circumferential elements from 40 to 64, and longitudinal elements from 22 to 38.

### Testing With Synthetic Data

Method accuracy was determined using idealized input data. Briefly, multiple simulations of diastolic inflation and systolic contraction for Patients 2 and 5 were firstly conducted by setting α to be 0 and 1, respectively (note that C_*H*_ and Tmax_*H*_ were set as the optimized values as presented in section “Testing With Synthetic Data” under “Results”). For each case, the simulated strain and LV end-systolic volume (ESV) were used as input in our optimization framework as “experimental data.” In addition, for Patient 2, the optimization was initiated with α set at 0, 0.5, and 1 while the *Tmax*_*H*_ was set at 100 kPa, 350 kPa, and 600 kPa to test whether the current optimization scheme is sensitive to the initial guess of the parameters.

### Virtual REVASC

Virtual REVASC was performed on patient-specific models that had an ischemia effect (α > 0) by setting α=0. As seen in Eq. 6, by setting α=0, *Tmax*_*n*_ becomes a function of LGE only and SP has no effect.

Virtual REVASC was validated by comparing sector specific radial strain, *E*_*rr*_ calculated from baseline models and after virtual REVASC with a Cine-CMR wall motion based estimation of revascularization effect. Briefly, the following relationship was developed:

(8) $$E_{r\,r}\,\_\,W\,M=E_{r\,r}\,\_\,B\,L\cdot \left(1+\Delta \,W\,M\cdot 50\%\right),$$

where *E_*rr*__BL* is *E*_*rr*_ calculated from baseline models, Δ*WM* the difference between Cine-CMR wall motion scores at baseline and after revascularization and *E_*rr*__WM* is an estimated *E*_*rr*_ after revascularization based on the change in wall motion score. Eq. 8 is consistent with a wall motion score of 0 having a wall thickening of at least 50%. For instance, if *E_*rr*__BL* = 0.2 and wall motion improved by 2 points, *E_*rr*__WM* would be 0.4.

## Results

Patient-specific LV and RV pressures and LV ejection fraction (EF) are shown in Table 1. Patient-specific LGE and SP scores at baseline and wall motion scores at baseline and follow-up studies are shown in Table 2. Four of the 5 patients had perfusion defects in sectors without MI/fibrosis (have over 3 sectors with SP score – LGE score >2). One of the patients had MI but no significant ischemia. Note that the wall motion scores were normal and LGE and SP scores were assumed = 0 for the healthy volunteer.

**TABLE 2:** Patient-specific LGE and SP scores at baseline (BL), and wall motion (WM) scores at BL and follow up (FU) studies.

**Subjects**	**Segments**	**1**	**2**	**3**	**4**	**5**	**6**	**7**	**8**	**9**	**10**	**11**	**12**	**13**	**14**	**15**	**16**	**17**
Patient 1	LGE score (BL)	0	0	0	3	1	1	0	0	0	3	4	2	0	0	0	0	0
	SP score (BL)	2	1	0	2	2	2	1	0	0	2	2	2	0	3	3	0	3
	WM score (BL)	1	1	1	3	4	3	1	1	1	3	4	0	0	0	0	0	0
	WM score (FU)	0	0	2	2	3	1	0	0	1	3	4	1	0	0	0	1	0
Patient 2	LGE score (BL)	0	0	0	0	3	0	1	0	0	1	3	1	1	0	0	0	0
	SP score (BL)	0	0	2	3	3	1	2	1	1	2	2	1	3	1	2	0	0
	WM score (BL)	0	0	1	3	4	1	3	0	0	3	3	1	3	0	3	1	3
	WM score (FU)	0	0	1	2	2	1	1	0	0	2	1	1	2	0	1	1	2
Patient 3	LGE score (BL)	0	0	0	0	0	0	0	0	0	1	1	0	0	0	0	0	0
	SP score (BL)	0	0	1	2	2	2	1	0	0	2	2	0	0	0	2	0	0
	WM score (BL)	3	3	3	4	3	3	3	3	3	4	3	3	3	3	4	3	3
	WM score (FU)	2	2	2	3	3	2	2	2	2	3	2	2	2	2	2	2	2
Patient 4	LGE score (BL)	0	0	0	0	0	0	1	1	0	0	0	0	2	1	0	0	3
	SP score (BL)	0	1	0	0	0	0	3	3	0	0	0	0	3	3	2	0	3
	WM score (BL)	0	0	0	0	0	0	1	0	0	0	0	0	3	3	1	0	4
	WM score (FU)	0	0	0	0	0	0	1	1	0	0	0	0	2	2	1	0	4
Patient 5	LGE score (BL)	0	0	0	0	4	0	0	0	0	1	4	0	1	0	0	3	0
	SP score (BL)	0	0	0	1	1	0	2	0	0	0	0	0	1	0	0	0	0
	WM score (BL)	0	0	0	0	2	0	0	0	0	1	3	1	0	0	1	3	0
	WM score (FU)	0	0	0	0	2	0	0	0	0	1	3	1	0	0	1	3	0
																		

### Mesh Convergence Study

The mesh convergence study determined that 7952 elements (Table 3) are required and further mesh refinement only results in an average of 2% change in *C*, < 2% change in *Tmax*_*H*_ and no change in α. Note that only the optimization results for using 7952 elements will be presented in the following sections.

**TABLE 3:** Mesh convergence study.

	**Number of elements**	**C_*H*_ (kPa)**	***Tmax*_*H*_ (kPa)**	**α**	**Calculation time (hrs×cpu)**
Patient 1	7952	0.099	523.3	1.0	137.3
Patient 2	2700	0.207	322.7	0.32	48.0
	4176	0.207	338.9	0.4	77.5
	7952	0.192	336.8	0.44	175.0
	10752	0.192	330.7	0.44	250.0
Patient 3	7952	1.7	173.5	0.08	599.4
Patient 4	7952	0.019	227.9	0	462.3
Patient 5	2700	0.0095	405.3	0.0	77.5
	4176	0.0095	414.8	0.0	125.0
	7952	0.0094	401.4	0.0	400.0
	10752	0.0090	408.6	0.0	560.0
Volunteer	7952	0.00011	259.4	N/A	1741.5

### Testing With Synthetic Data

**FIGURE 4:**
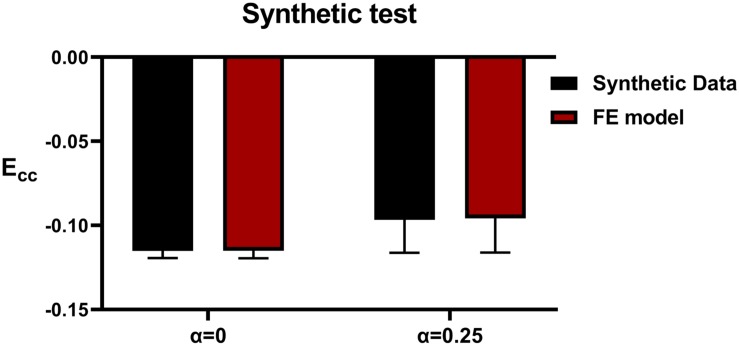
Comparison of synthetic *E*_*cc*_ with FE model predicted strain in patients 2 and 5.

Figure 4 shows excellent agreement between the synthetically generated “experimental strain” and the strain after optimization. The target and optimized parameters are summarized in Table 4 with a maximal difference of 1.4%, which shows the proposed method has great capability of predicting Tmax_*H*_ and α accurately. The synthetic test also demonstrated that the current optimization scheme is insensitive to the selection of the initial guess of the parameters.

**TABLE 4:** Results of testing with synthetic data.

**ID**	**Input**				**Optimized**	
	***Tmax*_*H*_ kPa)**	**α**	**Initial guess *Tmax*_*H*_ kPa)**	**Initial guess α**	***Tmax*_*H*_ kPa)**	**α**
Patient 2	336.8	0.0	100.0	0.0	336.7	0.0
			350.0	0.5	337.6	0.0
			600.0	1.0	336.7	0.0
Patient 2	336.8	1.0	100.0	0.0	332.3	1.0
			350.0	0.5	332.2	1.0
			600.0	1.0	332.6	1.0
Patient 5	401.4	0.0	350.0	0.5	401.5	0.0
Patient 5	401.4	1.0	350.0	0.5	401.0	1.0
						

### Prediction of *C*_*H*_, Tmax_*H*_ and α

*C*_*H*_ was determined such that the FE model predicted LV EDV matched the patient-specific *in vivo* measured EDV (Table 5).

**TABLE 5:** CMR-based *in vivo* volumes and the FE-predicted volumes after optimization.

**ID**	**CMR-based**		**FE-predicted**	
	**EDV (ml)**	**ESV (ml)**	**EDV (ml)**	**ESV (ml)**
Patient 1	245.4	157.0	245.4	158.3
Patient 2	160.9	111.0	160.9	108.3
Patient 3	300.0	255.5	300.0	249.2
Patient 4	170.0	97.4	170.0	96.5
Patient 5	94.9	52.2	94.9	51.3
Volunteer	118.7	49.7	118.7	48.6

**FIGURE 5:**
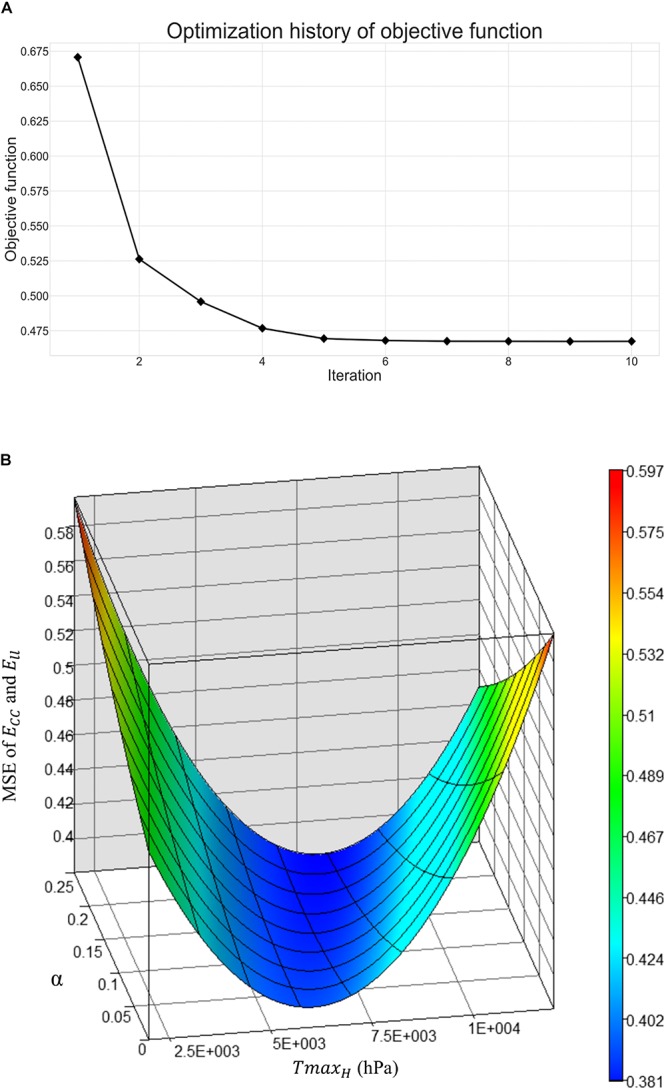
**(A)** Optimization history of the overall objective function, and **(B)** surface plot of mean-squared-error of strain with respect to *Tmax*_*H*_ and α.

**FIGURE 6:**
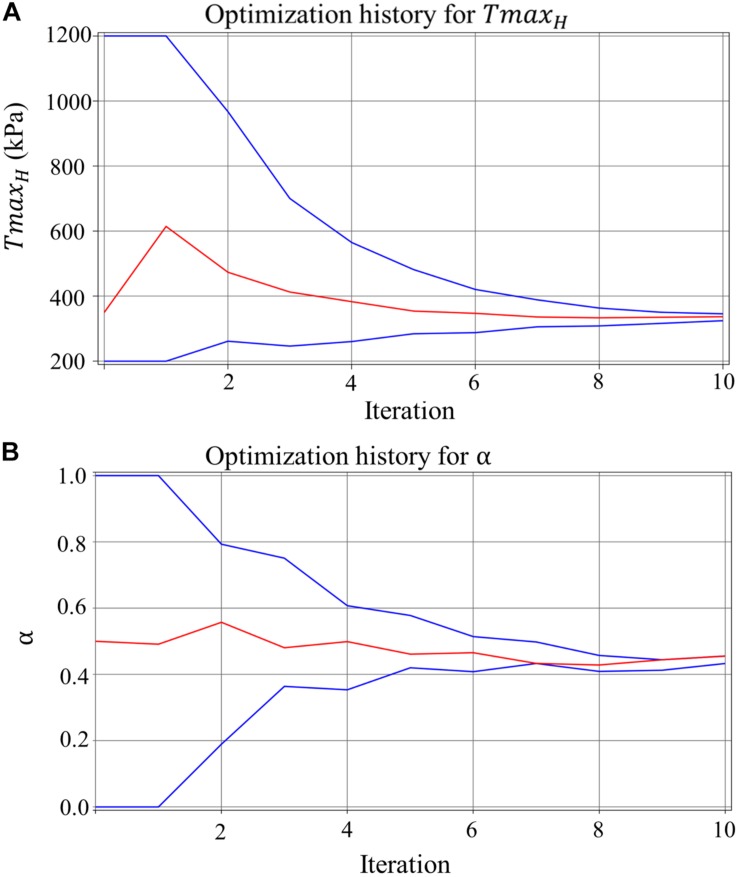
The convergence of the parameter (lines in blue represent the upper and lower bounds) for **(A)** *Tmax*_*H*_ for healthy myocardium, and **(B)** ischemia effect, α, on the LV myocardial contractility, with each parameter resulted in a precise final converged optimum.

Figure 5A shows excellent convergence of the objective function (OF) during a representative model optimization. Figure 5B shows a surface plot of MSE of the strain with respect to *Tmax*_*H*_ and α, where the valley of the surface indicates lowest MSE value relating to the best match between the experimental and FE model-predicted strain. The optimized *Tmax*_*H*_ and α are summarized in Table 3. The optimized *Tmax*_*H*_ and α displayed good convergence as shown in Figure 6. The final OF value of 0.297 (an average calculated using all six subjects) was obtained indicating generally good agreement between the FE model-predicted systolic strain and the patient-specific *in vivo* measured strain. The LV EDV and ESV were summarized in Table 5, where the results showed that the difference between the predicted LV ESV and volume measured with CMR was on average less than 2%.

**FIGURE 7:**
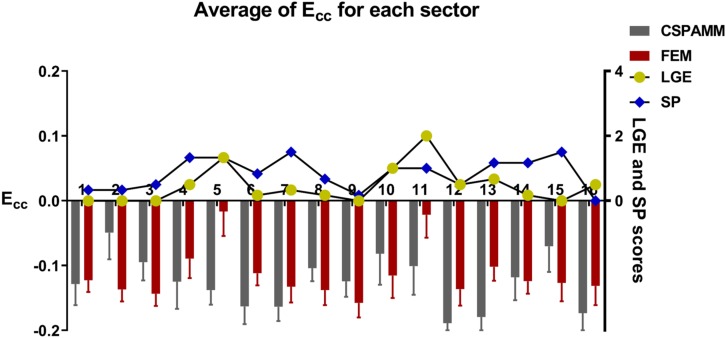
Comparison of sector-specific CSPAMM and FE model predicted *E*_*cc*_ across all 6 subjects. *E*_*cc*_ derived from CSPAMM and calculated from FE simulations are in gray and red bars, respectively. The average sector specific MI/fibrosis (LGE) and stress perfusion (SP) scores are in yellow circle and blue diamond, respectively.

The average of *E*_*cc*_ along with its standard error of the mean for all sectors with LGE score <3 from overall subjects derived from the experimental data and calculated with the FE model are −0.135 ± 0.044 and −0.127 ± 0.042. Figure 7 shows the regional comparisons of *E*_*cc*_ calculated from CSPAMM and FEM simulations. It can be seen that the model-predicted *E*_*cc*_ generally has good agreement with the *E*_*cc*_ derived from CSPAMM.

### Virtual REVASC

**FIGURE 8:**
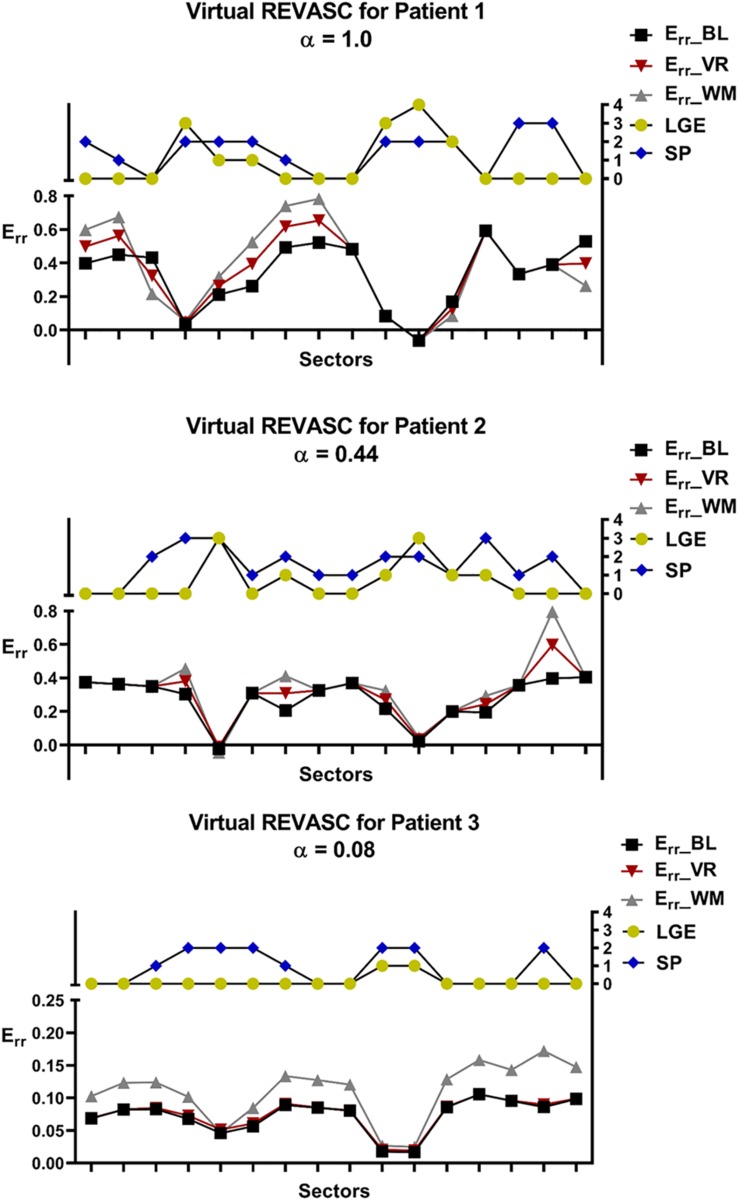
Comparison of the sector specific *E*_*rr*_ before and after virtual REVASC for the 3 patients with positive ischemia effect, α. Sector specific MI/fibrosis (LGE) and stress perfusion (SP) scores are plotted for reference. *E*_*rr*_ BL represents *E*_*rr*_ calculated using the FE simulations at baseline, *E*_*rr*_ VR is the *E*_*rr*_ obtained from the FE simulations after virtual REVASC, and *E*_*rr*_ WM is an “actual” *E*_*rr*_ effect estimated using the qualitative wall thickening scores.

Figure 8 shows the *E*_*rr*__*BL*, *E*_*rr*__*VR* and *E*_*rr*__*WM* for all 3 patients that were determined with positive α. In all studies, virtual REVASC underestimated the actual *E*_*rr*_ effect. In patients 1 and 2 the difference was mild. However, patient 3 had a small predicted alpha (0.08) and minimal improvement in *E*_*rr*_ with virtual REVASC in spite of the fact that at baseline there was significant ischemia and the improvement in actual wall motion after revascularization was significant.

## Discussion

In this study, we demonstrated a novel method to estimate the effect of ischemia on regional LV myocardial contractility. The proposed method takes the advantage of multiple CMR sequences and the FE modeling to formally optimize the LV mechanics parameters. To the best of our knowledge, this study is the first study that incorporated 17-segment-based patient-specific perfusion and LGE scores into constitutive model, which allowed regional mechanical properties to be estimated with better physiological constraints. A linear relationship was proposed to describe the effect of LV infarct and ischemia on LV active contraction. Our approach was tested with CMR data from five patients with multi-vessel CAD and moderate FMR and a healthy volunteer. Good agreement between the FE model-predicted systolic strains and the patient-specific *in vivo* measured strains were observed, which suggests that the optimized model was faithful to the experimental data.

### Ischemia Effect

The effect of ischemia, α, on LV contractility was found to be >0 for Patients 1, 2, and 3, but = 0 for Patients 4 and 5. This is consistent with the patient-specific wall motion scores seen in Table 2 where the cumulative wall motion score in patients 1, 2 and 3 decreased (improved) after PCI but remained unchanged or increased (worsened) in patients 4 and 5.

It should be noted that stress induced perfusion defects are associated with either normal or depressed regional myocardial function and in the latter case the myocardium would be described as hibernating (Wijns et al., 1998). First, by definition, our method identifies only hibernating myocardium and simple demand ischemia without dysfunction is not considered. As a corollary, if a patient with multi-vessel CAD had a large amount of demand ischemia without LV dysfunction, our method would not identify an ischemia effect. Specifically, if the SP defect is showing simple demand ischemia without underlying contractility deficit, then α would likely be 0. If the SP defect is associated with hibernating myocardium where contractility is reduced, then α will likely be >0.

### MI Stiffness

Our assumption that infarct stiffness is ten times that of normal myocardium is consistent with our prior studies (Walker et al., 2005; Wenk et al., 2011) and others (Genet et al., 2015). However, there is reason to believe that better measurement of infarct stiffness is necessary in patients with multi-vessel coronary disease and FMR. First, model accuracy was much better when sectors with transmural infarction were excluded. Second, the optimized *Tmax*_*H*_ was higher in Patients 1, 2, and 5 (523.3, 336.8, and 401.4 kPa respectively) than the healthy volunteer (259.4 kPa). The reason for this discrepancy is unclear but might be explained if Eq. 6 is overestimating the effect of infarct and ischemia on LV contractility. It is anticipated that future studies that measure infarct stiffness in patients with MI but without ischemia will help to better determine the relationship between infarct and myocardial contractility.

### Diastolic Stiffness in Patients With Multi-Vessel CAD

As seen in Table 3, there is a large variance in optimized *C*_*H*_ between the healthy volunteer and patients with multi-vessel CAD and FMR. We suspect this is likely a function of myocardial fibrosis (LGE scores >0) and, consistent with this, prior clinical studies have shown the extent of LGE to correspond to impaired LV relaxation (Moreo et al., 2009). However, the cumulative LGE scores of patients 1 and 2 are similar and further studies are therefore necessary to establish our hypothesis. On the other hand, the lack of diastolic strain in this study limits our ability to better optimize passive LV material parameters.

It is interesting to note that in Figure 7 in the sectors with large LGE scores (i.e., sectors 5 and 11), the model-predicted *E*_*cc*_ is much smaller than that derived from CSPAMM, while this is not seen in the sectors with large SP scores (i.e., sectors 4 and 7). This may be due to the effect of MI fibrosis/LGE on either regional diastolic stiffness or systolic contractility or interaction between the two. In the current study we assumed that systolic contractility was affected by both LGE and SP scores but that diastolic stiffness was only affected by the LGE scores. Those assumptions about the form and amount of LGE effect on diastolic stiffness and systolic contractility may be overestimated. Furthermore, it should be noted that fibrosis (LGE) -based effect on diastolic stiffness will affect systolic *E*_*cc*_ through its effect on strain at ED and sarcomere stretch. The mismatch between the model-predicted *E*_*cc*_ and *E*_*cc*_ derived from CSPAMM in the regions with large LGE scores shows that a better understanding of the effect of LGE scores on the diastolic stiffness is important.

### Model Accuracy

Results of a mesh sensitivity study suggest that the optimized LV material parameters are relatively insensitive to the mesh density, although slight differences were observed between FE models with different mesh density. Table 2 indicates that a mesh with 7952 elements may be the optimal choice for the current study because of the balanced calculation time and parameter optimization accuracy. Furthermore, synthetic test results suggest that the proposed method is capable of precisely determining the LV passive and active material properties and the ischemia effect on LV active contractility.

### Virtual REVASC

Validation of virtual REVASC using Cine-CMR wall thickening shows that the magnitude of ischemia effect is underestimated. This could be due to an incorrect *T*_*max*_ and LGE relationship. For example, we assumed a sector with LGE = 2 had the *T*_*max*_ as 50% of the *T*_*max*_ for a healthy sector and this may be over-estimating the effect of infarct on the myocardial contractility. As a result, according to Eq. 6, the model-predicted ischemia effect might be smaller than reality to compensate the over-estimated LGE effect on myocardial contractility. A non-linear relationship between *T*_*max*_ and LGE and a scaling factor in front of LGE score is under development.

Figure 8 shows that patient 3 had a small predicted alpha (0.08) and minimal improvement in *E*_*rr*_ with virtual REVASC in spite of the fact that at baseline there was significant ischemia and the improvement in actual wall motion after revascularization was significant. The reason for this is unclear but possibilities include that measured CSPAMM strain was inaccurate. In addition, patient 3 had a dilated LV and the subsequent decrease in systolic strain magnitude might make it hard to discriminate the ischemia (SP) effect. Regardless, this points out the need for accurate strain measurement with good spatial resolution as simulation input.

### Limitations

First, only systolic strains were calculated since the CSPAMM data were acquired during the systolic phase, which resulted in a fact that only systolic myocardial material parameters can be optimized. Because of the lack of diastolic strains, the main diastolic material parameter, *C*_*H*_ was calibrated to match the measured LV end-diastolic volume. Second, the border zone effect was not taken into consideration in the current study. Third, only a linear relationship between the infarct and ischemia and the LV contractility were employed in this study. Fourth, boundary conditions near the valve plane of the LV may affect longitudinal strain calculations in that local area. However, longitudinal strain in the body of the LV is realistic. Last, the path from end-diastole to end-systole is not specified, and our models cannot therefore provide accurate simulation of isovolumic contraction and ejection. However, simulation of diastolic filling and end-systole are accurate.

## Conclusion

This study proposed a novel FE method to predict the effect of ischemia on the LV contractility in patients with multi-vessel coronary disease and FMR. The proposed method has good agreement with patient-specific *in vivo* measured strains and is able to predict REVASC effect.

Future studies will explore the stress perfusion and contractility relationship to determine if non-linear relationships allow greater model accuracy. Flow structure models will be implemented so that mitral regurgitation effect can be directly simulated. Last, a study with larger numbers of patient-specific models will better establish the utility of our FE method.

## Data Availability Statement

All datasets generated for this study are included in the article/supplementary material.

## Ethics Statement

The Cornell Institutional Review Board approved this study, and written informed consent was obtained at the time of enrollment.

## Author Contributions

YZ, JW, and MR designed the study. YZ conducted simulations, analyzed results, and wrote the initial draft of the manuscript. MR created the FE models. VW, AM, and JK did data analysis. LG contributed to development of methodology. JW collected the clinical data. LG, JW, and MR supervised the study. YZ, VW, JG, JW, and MR contributed to manuscript writing/review.

## Conflict of Interest

The authors declare that the research was conducted in the absence of any commercial or financial relationships that could be construed as a potential conflict of interest.

## Footnotes

1 https://github.com/mratcliffe/ElementSearch/, available on request
